# Electrocardiographic Markers Indicating Right Ventricular Outflow Tract Conduction Delay as a Predictor of Major Arrhythmic Events in Patients With Brugada Syndrome: A Systematic Review and Meta-Analysis

**DOI:** 10.3389/fcvm.2022.931622

**Published:** 2022-06-17

**Authors:** Mohammad Iqbal, Iwan Cahyo Santosa Putra, Raymond Pranata, Michael Nathaniel Budiarso, Miftah Pramudyo, Hanna Goenawan, Mohammad Rizki Akbar, Arief Sjamsulaksan Kartasasmita

**Affiliations:** ^1^Department of Cardiology and Vascular Medicine, Faculty of Medicine University of Padjadjaran, Bandung, Indonesia; ^2^School of Medicine and Health Sciences, Atma Jaya Catholic University of Indonesia, Jakarta, Indonesia; ^3^Division of Physiology, Department of Biomedical Sciences, Faculty of Medicine University of Padjadjaran, Bandung, Indonesia; ^4^Faculty of Medicine University of Padjadjaran, Bandung, Indonesia

**Keywords:** Brugada syndrome, RVOT conduction delay sign, aVR sign, S wave in the lead I, major arrhythmic events

## Abstract

**Introduction:**

Risk stratification in Brugada Syndrome (BrS) patients is still challenging due to the heterogeneity of clinical presentation; thus, some additional risk markers are needed. Several studies investigating the association between RVOT conduction delay sign on electrocardiography (ECG) and major arrhythmic events (MAE) in BrS patients showed inconclusive results. This meta-analysis aims to evaluate the association between RVOT conduction delay signs presented by aVR sign and large S wave in lead I, and MAE in BrS patients.

**Methods:**

The literature search was performed using several online databases from the inception to March 16^th^, 2022. We included studies consisting of two main components, including ECG markers of RVOT conduction delay (aVR sign and large S wave in lead I) and MAE related to BrS (syncope/VT/VF/SCD/aborted SCD/appropriate ICD shocks)

**Results:**

Meta-analysis of eleven cohort studies with a total of 2,575 participants showed RVOT conduction delay sign was significantly associated with MAE in BrS patients [RR = 1.87 (1.35, 2.58); *p* < 0.001; *I*^2^= 52%, *P*_heterogeneity_ = 0.02]. Subgroup analysis showed that aVR sign [RR = 2.00 (1.42, 2.83); *p* < 0.001; *I*^2^= 0%, *P*_heterogeneity_ = 0.40] and large S wave in lead I [RR = 1.74 (1.11, 2.71); *p* = 0.01; *I*^2^= 60%, *P*_heterogeneity_ = 0.01] were significantly associated with MAE. Summary receiver operating characteristics analysis revealed the aVR sign [AUC: 0.77 (0.73–0.80)] and large S wave in lead I [AUC: 0.69 (0.65–0.73)] were a good predictor of MAE in BrS patients.

**Conclusion:**

RVOT conduction delay sign, presented by aVR sign and large S wave in the lead I, is significantly associated with an increased risk of MAE in BrS patients. Hence, we propose that these parameters may be useful as an additional risk stratification tool to predict MAE in BrS patients.

**Systematic Review Registration:**

https://www.crd.york.ac.uk/prospero/#recordDetails, identifier: CRD42022321090.

## Introduction

Brugada syndrome (BrS) is an inherited channelopathy that predisposes to sudden cardiac death (SCD) due to ventricular tachyarrhythmias (VTA) (1). The prevalence of BrS was 0.5 per 1,000 populations around the globe and accounted for 4 per cent of SCD (2, 3). Although BrS has a low prevalence, the risk of SCD caused by VTA in BrS patients with a structurally normal heart remains high, up to 20% (4). Shanghai et al. (5) and Sieira et al. (6) scores, which were established previously to diagnose BrS, have been used recently as stratification risk scores to predict SCD (7). The components of these two scores are type 1 Brugada electrocardiographic (ECG) pattern, syncope or unexplained cardiac arrest history, family history of SCD or confirmed BrS, and positive genetic result (5, 6). However, until now, risk stratification in BrS patients is still challenging because of a dynamic ECG pattern of Brugada, heterogeneity of clinical presentation, and unidentified clinical history.

Furthermore, in some cases, the decision of ICD implantation may be challenging, especially in BrS type I pattern on ECG with unexplained syncope (ex; syncope due to cardiac vs. non-cardiac) and without prior cardiac arrest or documented VTA. Hence, several studies investigated the association between several ECG markers and major arrhythmic events (MAE) in BrS patients to predict the likelihood of SCD and VTA events. Several meta-analyses revealed that first-degree atrioventricular block, fragmented QRS, wide QRS complex, early repolarization, especially in the inferolateral region, atrial fibrillation, Tpeak-Tend dispersion, Tpeak-Tend interval, and (Tpeak-Tend)/QTc ratio were significantly associated with a higher risk of MAE in BrS patients (8–14).

Studies by Coronel et al. (15) and Panonne et al. (16–18) also discovered the right ventricular outflow tract (RVOT) conduction delay as the origin of VTA in BrS patients. Therefore, hypothetically, RVOT conduction delay sign may predict the VTA events in BrS patients. RVOT conduction delay can be represented by two ECG findings, a positive aVR sign and a large S wave in the lead I (19). Several observational studies investigated the association between RVOT conduction delay sign on ECG and MAE in patients with BrS, but the results were equivocal (20–24). Hence, this meta-analysis aims to systematically evaluate the association between RVOT conduction delay presented by aVR sign and large S wave in the lead I with MAE in patients with BrS.

## Methods

This meta-analysis adhered to the Preferred Reporting Items for Systematic Reviews and Meta-analysis (PRISMA) guideline.

### Search Strategy

We conducted a systematic literature search from several databases, including Pubmed, EBSCO host, Europe PMC, and Proquest. The keyword of “(aVR sign or S wave in the lead I) AND (major arrhythmic events or syncope or ventricular tachyarrhythmia or ventricular tachycardia or ventricular fibrillation or sudden cardiac death or aborted sudden cardiac death or appropriate implantable cardioverter-defibrillator shocks) AND (Brugada syndrome)” was used. The timeframe was from the inception until March 16^th^, 2022. Two independent authors performed title or abstract screening and eligibility assessment of the articles. Discrepancies were resolved by discussion.

### Eligibility Criteria

Studies that met the following criteria were included: prospective or retrospective cohort studies reporting ECG findings of RVOT conduction delay and MAE in BrS patients. BrS is diagnosed in patients with typical type 1 Brugada ECG patterns that occur spontaneously or drug-induced (25). There were two ECG patterns indicating RVOT conduction delay evaluated in this study, including a positive aVR sign and a large S wave in the lead I. The positive aVR sign is defined as R wave amplitude ≥0.3 mV in lead aVR or R/q ≥ 0.75 in lead aVR (20–22). While the criteria diagnosis of large S wave in the lead I is S wave amplitude ≥0.1 mV and/or S wave duration >40 ms in the lead I (22–24, 26–29). The outcome of interest in this study was MAE, including syncope, ventricular tachycardia (VT), ventricular fibrillation (VF), sudden cardiac death (SCD), aborted SCD, and appropriate implantable cardioverter-defibrillator (ICD) shocks. Syncope is defined as a patient with loss of consciousness that is expectingly caused by VTA and after excluding other possible causes, including neurally mediated syncope (23). VT and VF are defined as wide QRS complex tachycardia, originally initiated from the ventricular wall. VT and VF were recorded at the follow-up period by ECG, Holter monitoring, or implantable cardioverter-defibrillator (ICD) (23, 26). SCD is defined as death most likely caused by an arrhythmic event, and no evident extra-cardiac causes were identified (25). Moreover, studies that met one of the following criteria were removed: (1) review articles, (2) editorial/commentaries, (3) abstracts, (4) letters, (5) case reports, (6) case-control studies, (7) cross-sectional studies, and (8) studies in languages other than English.

### Data Extraction and Quality Assessment

Two independent authors performed data extraction of the eligible studies using standardized extraction form for the first author, study design, location of the study, inclusion criteria, sample size, age, sex, male gender, RVOT conduction delay sign on ECG and its criteria, mean/median duration of follow up and the outcomes. Any discrepancies were resolved by discussion.

Two independent authors performed the risk of bias assessment using the Newcastle-Ottawa Scale (NOS) (30). Scoring for every article was assigned based on its degree of bias [low (included) and high (excluded)]. If a study received a total score equal to or >7, it indicated a good journal quality with a low risk of bias. Otherwise, if a study received a total score of six or less, it was determined to have a significant risk of bias and was eliminated from the study selection process. Discrepancies during the risk of bias assessment were resolved by discussion with the third reviewer.

### Statistical Analysis

All statistical analysis was performed using Review Manager software version 5.4.1 (Cochrane Collaboration) and STATA software version 16. We calculated the pooled risk ratio (RR) and its 95% confidence interval using the Mantel-Haenzel formula to characterize the association between ECG findings of RVOT conduction delay and MAE in patients with BrS. The significance was obtained if the two-tailed *p*-value was ≤ 0.05. Inconsistency index (*I*^2^) test ranging from 0 to 100% was used to assess heterogeneity among the studies, in which *I*^2^ values >50% or *P*_heterogeneity_ < 0.05 indicate moderate to high heterogeneity (31). If high heterogeneity was found, a random-effects model was assigned to calculate the pooled RR. Otherwise, a fixed-effects model was used if low heterogeneity was noted. We performed a sensitivity analysis by excluding the study by leaving one method to detect the source of heterogeneity and obtained statistical robustness. Moreover, we performed a sub-group analysis comprising two ECG patterns of RVOT conduction delay to compare which ECG patterns were more significantly associated with MAE in BrS patients. We also performed a summary receiver operating characteristics (SROC) analysis to assess the predictive accuracy of these ECG markers in predicting MAE in BrS patients. Lastly, Begg's funnel plot and Egger's test were employed to discover publication bias and small-study effects.

## Results

### Characteristics of Included Studies

**Figure 1:**
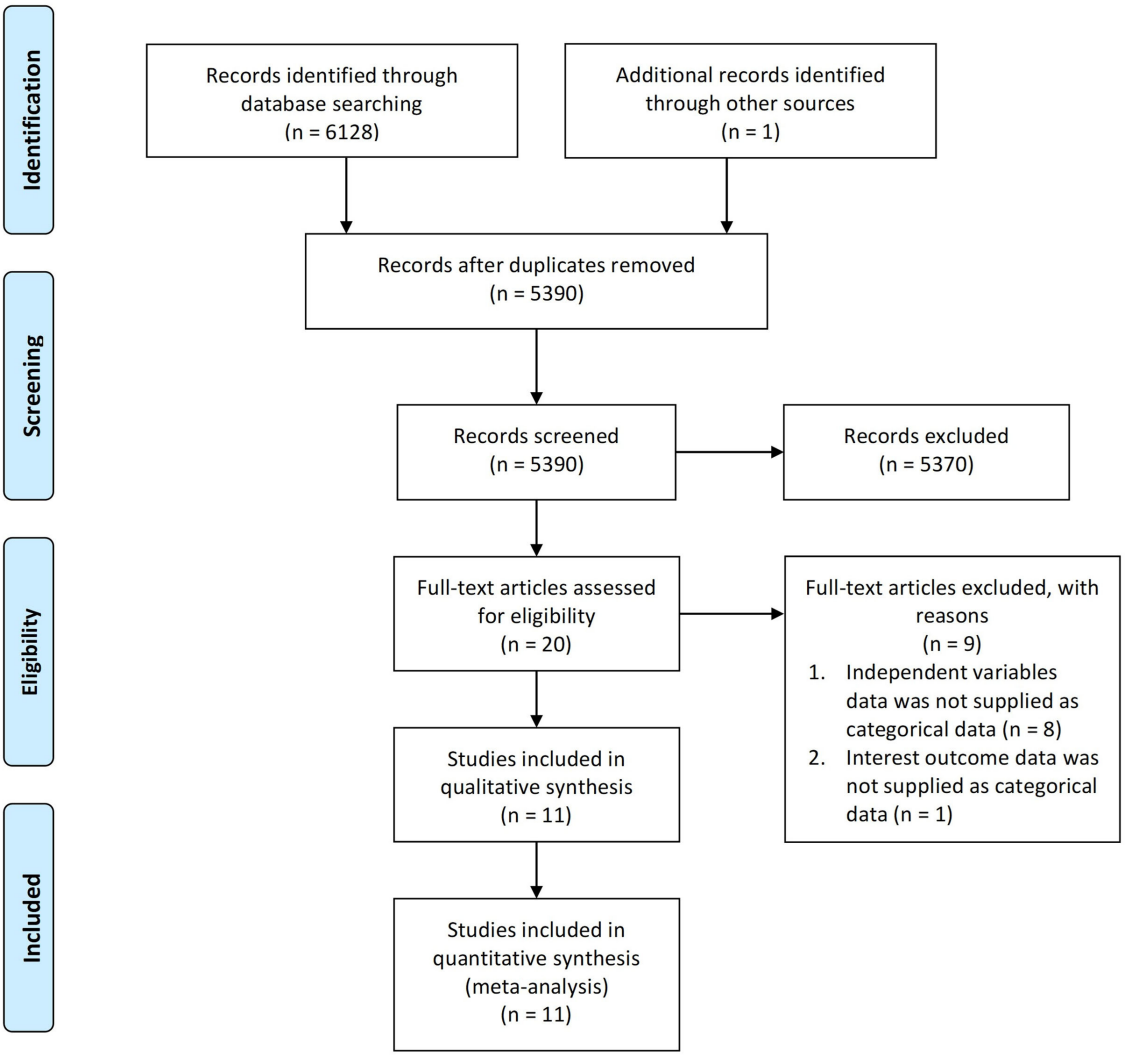
PRISMA flowchart.

A total of 6,129 articles were collected from the literature search, one of which was discovered via the hand-searching procedure and 5,390 of which remained after duplicates were removed. Following a screening procedure for titles and abstracts, 20 suitable studies were retained. Furthermore, 9 studies were excluded based on two reasons: (1) independent variables were not supplied in categorical data (*n* = 8), and (2) outcomes of interests were not supplied in categorical data (*n* = 1). Eventually, at the end of the preliminary search, 11 cohort studies (20–24, 26–29, 32, 33) were included in the qualitative analysis. Bias risk quality assessment was performed using the New Castle Ottawa Scale (NOS), resulting in 5 studies receiving a score of 7 and 6 studies receiving a score of 8, which indicates that all included studies were good quality journals with a low risk of bias (Supplementary Table 1). Hence, these 11 cohort studies with a total of 2,575 participants were included in the quantitative analysis. The PRISMA flowchart describes the selection process as illustrated in Figure 1.

Our meta-analysis consisted of two studies (23, 29) using a prospective design, while the rest used a retrospective design (20–22, 24, 26–28, 32, 33). Most of the participants were male (77.5%), and there were two studies (20, 22) where all the subjects were male. There were two types of ECG parameters indicating RVOT conduction delay, which were investigated in these included studies, including a positive aVR sign and a large S wave in the lead I. The number of participants who had positive aVR sign and large S wave in the lead I were 16.3 and 36.3%, respectively (20–22, 26). The total MAE calculated for all participants in all included studies was 15.9%. The characteristic of included studies is described in Table 1.

**Table 1:** The characteristic of included studies.

**No**	**Reference**	**Country**	**Study design**	**Sample size (n)**	**Age (mean ±SD)**	**Male n (%)**	**RVOT conduction delay sign on ECG**	**ECG criteria**	**Mean/median duration of follow up (months)**	**Outcomes**	**NOS**
1	Bigi et al. (20)	Iran	Retrospective cohort study	24	31 ± 7.5	24 (100)	aVR sign	R wave amplitude ≥ 0.3 mV or R/q ratio ≥ 0.75 in lead aVR	50	Syncope/VT/VF/ aborted SCD	7
2	Calo et al. (23)	Italy	Prospective cohort study	347	45 ± 13.1	272 (78)	Significant S wave in lead I	S wave amplitude ≥ 0.1 mV and duration ≥ 40 ms in lead I	48	VT/VF/SCD/ aborted SCD	8
3	Ragab et al. (21)	Netherlands	Retrospective cohort study	132	43 ± 15	86 (65)	aVR sign	R wave amplitude ≥ 0.3 mV in lead aVR	44	VT/VF	8
4	Ragab et al. (33)	Netherlands	Retrospective cohort study	147	43 ± 15	97 (65)	Large S wave in lead I	S amplitude ≥ 0.15 mV	56	VT/VF	8
5	Morita et al. (22)	Japan	Retrospective cohort study	62	NA	62 (100)	aVR sign and large S wave in lead I	R wave amplitude > 0.3 mV or R/q ratio of ≥ 0.75 in lead aVR S wave amplitude ≥ 0.1 mV and/or duration ≥ 40 ms	48	VF	7
6	Rizal et al. (24)	Indonesia	Retrospective cohort study	22	NA	19 (86)	large S wave in lead I	S wave amplitude ≥ 0.1 mV and duration ≥ 40 ms in lead I	27	VT/VF/ICD shocks	7
7	Honarbakhsh et al. (26)	Multicenter international	Retrospective cohort study	1110	51.8 ± 13.6	790 (71.2)	aVR sign and significant S wave in lead I	R wave amplitude ≥ 3 mm or R/q ratio ≥ 0.75 in lead aVR S wave amplitude of ≥0.1 mV in lead I	64	VT/VF/aborted SCD/ICD shocks	8
8	Nagase et al. (27)	Japan	Retrospective cohort study	209	45 ± 14	200 (96)	Prominent S wave in lead I	S-wave amplitude ≥ 0.1 mV and/or duration ≥ 40 ms in lead I	56	VF	8
9	Shinohara et al. (32)	Japan	Retrospective cohort study	193	50 ± 13	180 (93)	Large S wave in lead I	Not mentioned in detail	101	VT/VF/ICD shocks	7
10	Michowitz et al. (28)	Multicenter international	Retrospective cohort study	57	14	42 (73)	Large S wave in lead I	S-wave amplitude ≥ 0.1 mV or duration ≥ 40 ms in lead I	39	Arrhythmic Events	7
11	Migliore et al. (29)	Italy	Prospective cohort study	272	43 ± 12	223 (82)	Large S wave in lead I	S-wave amplitude ≥ 0.1 mV and/or duration > 40 ms in lead I	85	SCD/VF/ICD shocks	8

*RVOT, right ventricular outflow tract; ECG, electrocardiographic; NOS, Newcastle Ottawa Scale; VT, ventricular tachycardia; VF, ventricular fibrillation; SCD, sudden cardiac death; ICD, implantable cardioverter defibrillator; ms, milliseconds*.

### Meta-Analysis of RVOT Conduction Delay Sign and Major Arrhythmic Events

**Figure 2:**
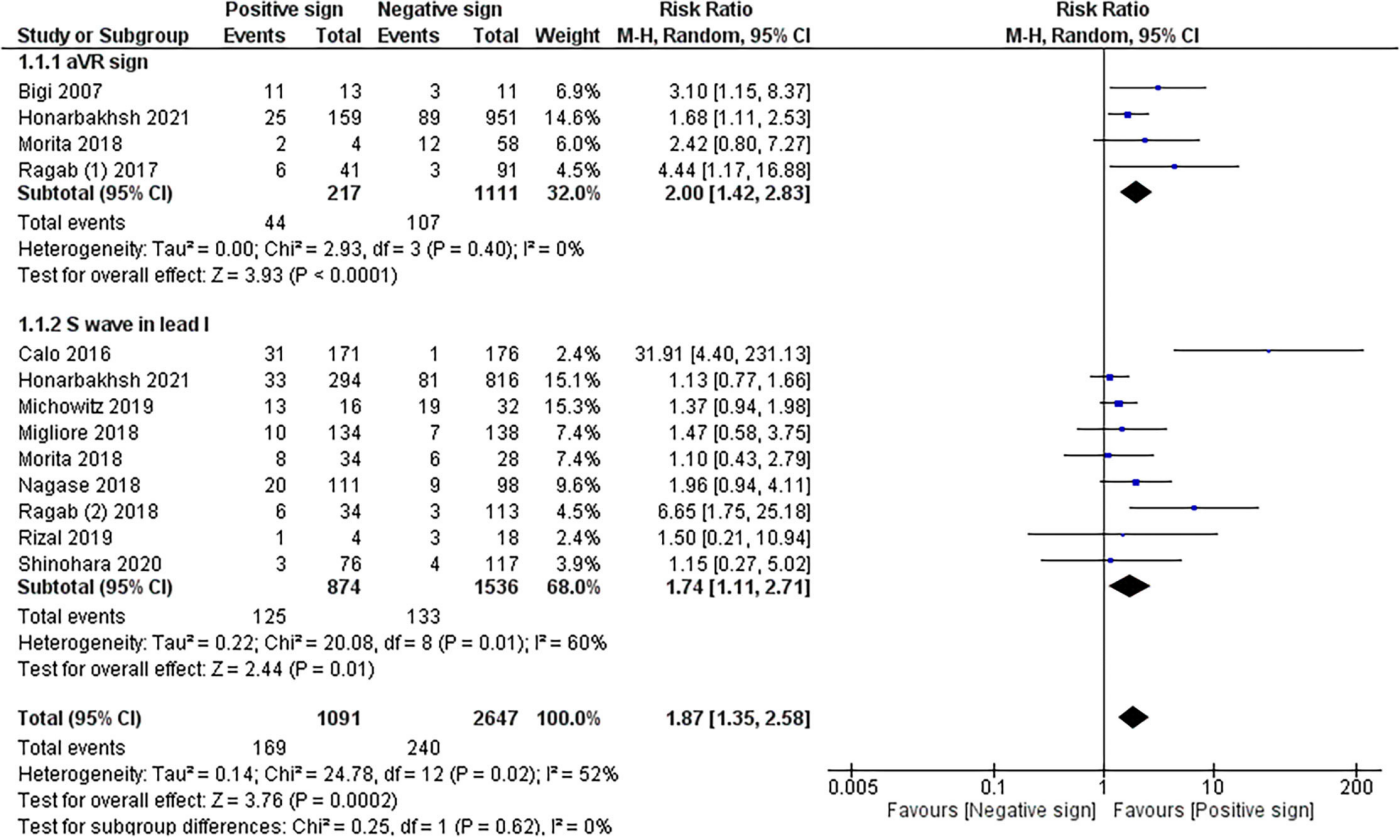
Meta-analysis of RVOT conduction delay on ECG and MAE in patients with Brugada syndrome.

The meta-analysis in 10 cohorts studies with pooled subjects of 2,575 participants showed that RVOT conduction delay sign presented by positive aVR sign and prominent S wave in lead I combined was significantly associated with MAE in patients with BrS (RR = 1.87 (1.35, 2.58); *p* < 0.001; *I* ^2^= 52%, *P*_heterogeneity_ = 0.02). The meta-analysis of ECG patterns of RVOT conduction delay and MAE in patients with BrS is described in Figure 2.

### Sub-group Analysis of aVR Sign and Major Arrhythmic Events

Sub-group analysis of 4 cohorts studies (20–22, 26) with pooled subjects of 1,328 participants revealed that aVR sign significantly increased the risk of MAE in BrS patients (RR = 2.00 (1.42, 2.83); *p* < 0.001; *I*^2^= 0%, *P*_heterogeneity_ = 0.40).

### Sub-group Analysis of Large S Wave in the Lead I and Major Arrhythmic Events

Sub-group analysis of 9 cohort studies (22–24) with pooled subjects of 2,410 participants showed that a large S wave in the lead I was significantly associated with a higher risk of MAE in BrS patients [RR = 1.74 (1.11, 2.71); *p* = 0.01; *I*^2^= 60%, *P*_heterogeneity_ = 0.01]. Due to moderate heterogeneity, sensitivity analysis was performed by excluding the Calo et al. study resulting in reduced heterogeneity to 8%, and the association remained significant [RR = 1.39 (1.08, 1.78); *p* = 0.01; *I* ^2^= 8%, *P*_heterogeneity_ = 0.37].

### SROC Analysis of RVOT Conduction Delay Sign in Predicting Major Arrhythmic Events

**Figure 3:**
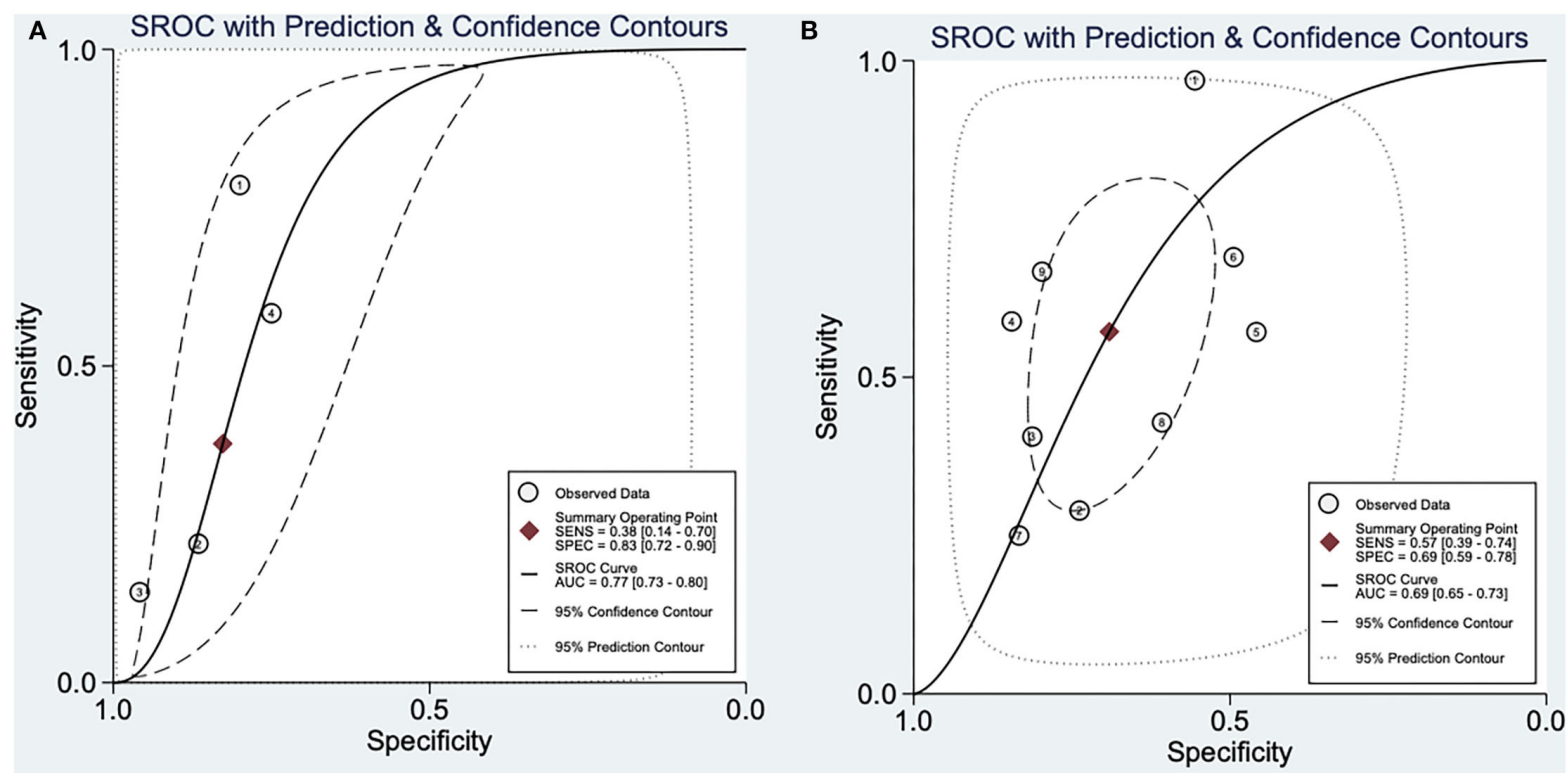
Summary receiver operatic characteristic analysis. **(A)** SROC analysis of aVR sign in predicting major arrhythmic events in BrS patients. **(B)** SROC analysis of large S wave in lead I in predicting major arrhythmic events in BrS patients. SROC, summary receiver operating characteristic; AUC, area under curve; SENS, sensitivity; SPEC, specificity.

SROC analysis showed that positive aVR sign on ECG had a great diagnostic performance in predicting MAE in BrS patients [AUC: 0.77 (0.73–0.80), sensitivity: 38%, specificity: 83%, positive likelihood ratio: 2.2 (1.4, 3.4), negative likelihood ratio: 0.75 (0.50, 1.13), diagnostic odds ratio: 3.0 (1, 7)]. On the other hand, large S wave in lead I on ECG was also a good predictor of MAE in BrS patients [AUC: 0.69 (0.65–0.73), sensitivity: 57%, specificity: 69%, positive likelihood ratio: 1.85 (1.3, 2.62), negative likelihood ratio: 0.62 (0.42,0.92), diagnostic odds ratio: 2.98 (1.47, 6.07)]. SROC analysis is described in Figure 3.

### Publication Bias

**Figure 4:**
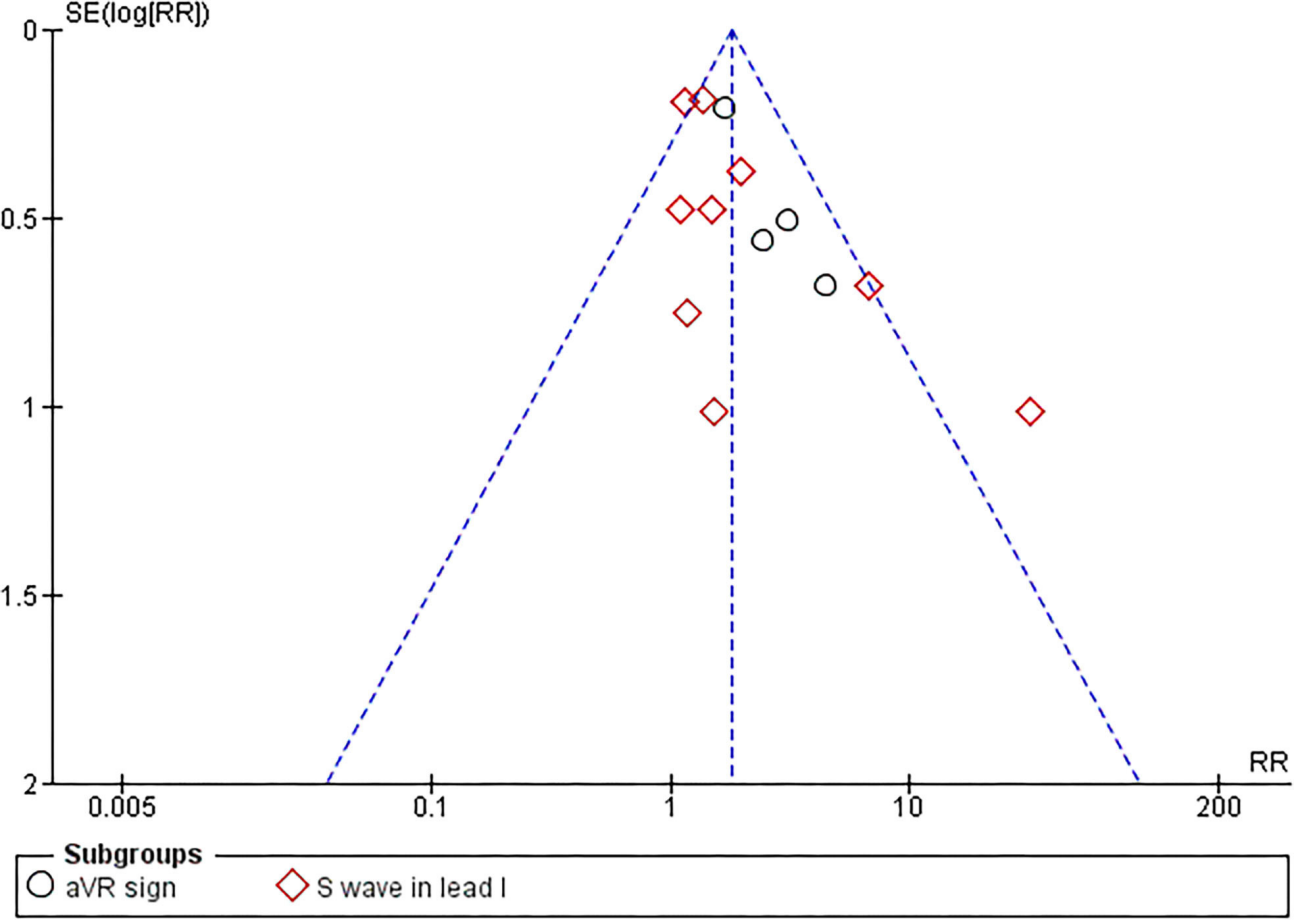
Begg's funnel plot.

Begg's funnel plot analysis revealed qualitatively symmetrical funnel plots, suggesting no evidence of publication bias in association between RVOT conduction delay sign and MAE (Figure 4). Moreover, Egger's test showed no evidence of small-study effects for RVOT conduction delay sign and MAE (*p* = 0.097).

## Discussion

To the best of our knowledge, this is the first meta-analysis to demonstrate the association between RVOT conduction delay on ECG and MAE in patients with BrS. There were several significant findings in our study. Firstly, the RVOT conduction delay sign on ECG was significantly associated with MAE in BrS patients. Secondly, a positive aVR sign and large S wave in the lead I were significantly associated with higher MAE risk in BrS patients.

Up until now, there are two widely accepted hypotheses related to the pathomechanism of BrS, including repolarization and depolarization abnormalities, which all originated from RVOT (17, 34). Pannone et al. performed a high-density RVOT epicardial electroanatomic mapping (EAM) analysis using an ajmaline test in BrS patients and showed the presence of high-frequency potentials (HFPs) in all patients before and after the ajmaline test, and low-frequency potentials (LFPs) in all patients after the ajmaline test. HFPs and LFPs, which were the expression of abnormal depolarization and repolarization, respectively, were correlated with RVOT conduction delay (17).

These RVOT conduction delay pathomechanism can also be elaborated in the case of widely known positive SCN5A mutation, which can increase the risk of SCD (5, 6). Another study by Pannone et al. (18) by utilizing electrocardiographic imaging (ECGI) after ajmaline administration revealed a significantly longer RVOT activation time (RVOT-AT) and RVOT recovery time (RVOT-RT) in BrS patients with positive SCN5A mutation compared to BrS patients without SCN5A mutation. According to EAM analysis combined with ajmaline administration, BrS patients with positive SCN5A mutation also had substantially higher HFP activation time (HFPat), LFPat, and LFP duration (LFPd), which concludes that BrS patients with SCN5A mutation exhibited RVOT conduction delay.

Furthermore, Pannone et al. (16) also evaluated the ECGI and EAM analyses in BrS patients with a history of aborted SCD, which showed a significantly higher RVOT-AT and lower RVOT activation-recovery interval (RVOT-ARI) in ECGI after ajmaline administration compared to patients without a history of aborted SCD. Consistently, EAM analysis confirmed that BrS patients with a history of aborted SCD had significantly higher HFPat, LFPat, and LFPd, indicating that RVOT conduction delay also plays an important role in the development of VTA in BrS patients (17). It appears that ajmaline, a sodium channel blocker agent, could unmask the covert electrical substrate that caused the RVOT conduction delay in BrS patients. Moreover, an experimental study conducted by Coronel et al. studied the heart structure of BrS patients who underwent heart implantation surgery and did not have prior structural heart disease. This study showed that the interstitial fibrotic process caused slow conduction in the RVOT area. Based on the activation mapping, this RVOT conduction delay was responsible for typical ECG changes in BrS patients and was the source of VTA (15). Therefore, based on all this evidence, identifying RVOT conduction delay signs may help predict MAE incidence in BrS patients.

A vectorcardiography (VCG) study in BrS patients showed that right terminal conduction delay (RECD) was detected in the upper right quadrant and posterior quadrant of the VCG (35). These VCG regions correspond to the aVR leads on the ECG and were anatomically opposite to the RVOT. Hence, the RVOT conduction delay produced a prominent R wave in lead aVR and a large S wave in the lead I due to its rightward direction of the depolarization wave. Although aVR sign and large S wave in lead I are good predictors of MAE, the prevalence of these ECG markers varied among BrS patients according to clinical presentation and age. Minier et al. (36) study showed that positive aVR signs commonly occurred in young BrS patients (under 17 years old) compared to middle-aged (17–59 years) and old (60 years and over) BrS patients. Moreover, Bigi et al. (20) study showed that BrS patients that previously experienced VTA were more likely to have higher R-wave amplitude or R/q ratio in lead aVR. Based on two cohort studies, positive aVR signs more frequently occurred in symptomatic patients than asymptomatic BrS patients (20, 21). In contrast, the other two cohort studies found that the incidence of positive aVR signs was not significantly different between symptomatic and asymptomatic BrS patients (37–39). On the other hand, three cohort studies found that large S wave in the lead I was not different between symptomatic and asymptomatic BrS patients (27, 37, 39). Nonetheless, Ragab et al. study revealed that large S wave in lead I was more likely occurred in symptomatic BrS patients. Thus, further cohort studies are needed to compare the impact of these ECG markers on MAE in BrS patients according to patients' age and clinical presentation.

Moderate heterogeneity was detected in the analysis of large S wave in the lead I with MAE; thereby, sensitivity analysis by excluding Calo et al. study was performed, resulting in significantly reduced heterogeneity to 8% and without altering the significance. It is due to Calo et al. study, which has a remarkably high-risk ratio (RR) compared to the other studies. The possible explanation was because the 5 per cent of BrS patients with large S wave in the lead I in Calo et al. study appeared to have an MAE on the same day or within days of their diagnosis (i.e., approximately one-third of all participants who had MAE during follow-up). It is questionable that MAE develops in such a short time after the initial diagnosis of BrS in the Calo et al. study. Nevertheless, it might be explained that all participants in this study had spontaneous type I Brugada ECG pattern and had most of the inducible VTA by electrophysiological study (EPS) compared to other included studies, which carried a high risk of MAE.

A retrospective study conducted by Pannone et al. (16) showed that several components of ECGI analysis after ajmaline induction, including RVOT-AT > 110.5 milliseconds, delta RVOT-AT > 40.3 milliseconds, RVOT-ARI < 267.5 milliseconds and delta RVOT-ARI < 18 milliseconds were good predictors of aborted SCD events in BrS patients with AUC value was 0.92, 0.86, 0.79, and 0.76, respectively. Compared to our results, the prognostic value of aVR wave sign and large S wave in lead I were inferior to RVOT-AT and delta RVOT-AT yet comparable to RVOT-ARI and delta RVOT-ARI. Although our result shows a modest AUC in both aVR and large S wave sign, we should underline the potential role of ECG to detect the RVOT conduction delay in daily practice as ECG could provide a more non-invasive characteristic, yet also still a convenient and affordable tool to be operated even in the most primary care. Hence, we may suggest the positive aVR sign and large S wave in lead I on ECG as an essential factor during risk stratification to predict MAE in BrS patients.

**Figure 5:**
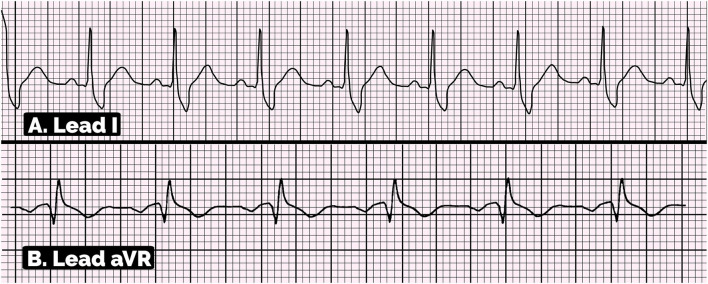
Electrocardiographic pattern of positive aVR sign and large S wave in lead I. **(A)** This ECG showed S-wave amplitude ≥0.1 mV and duration >40 ms in lead I, suggesting the large S wave in lead I. **(B)** This ECG showed the positive aVR sign with the amplitude of R wave in aVR lead is ≥0.3 mV and R/q ratio is ≥0.7.

The positive aVR sign and large S wave in the lead I on ECG are described in Figure 5. The diagnosis criteria of aVR sign are R wave amplitude ≥ 0.3 mV in lead aVR or R/q ≥ 0.75 in lead aVR (20–22). On the other hand, the criteria for diagnosis of S wave in the lead I are S-wave amplitude ≥ 0.1 mV and/or duration >40 ms in the lead I (23, 26–29, 37).

This meta-analysis has several limitations. Firstly, most of the included studies were retrospective designs, increasing the risk of recall and selection bias. Secondly, several confounding factors, including ECG pattern of spontaneous type I Brugada, prior syncope, family history of SCD, presence of SCN5A gene mutation, and inducible VTA in EPS, were not adjusted in most included studies, possibly leading to an increased risk of bias. Thirdly, high heterogeneity was noted in a sub-group analysis of large S wave in the lead I, which was likely caused by differences in included participants' characteristics in the Calo et al. study. Lastly, due to the lack of studies investigating the impact of ECG parameters indicating RVOT conduction delay sign on MAE in BrS patients according to patients' age and clinical presentation, further prospective cohort studies are still needed to understand this ECG pattern in BrS patients better.

## Conclusion

In conclusion, this meta-analysis shows that the RVOT conduction delay sign, including the positive aVR sign and large S wave in the lead I, is significantly associated with MAE in patients with BrS. Furthermore, the aVR sign and large S wave in lead I can be used as a potential ECG marker to predict MAE. Further prospective cohort studies are still needed to establish the association between these ECG parameters and MAE in BrS patients in the near future.

## Data Availability Statement

The original contributions presented in the study are included in the article/Supplementary Material, further inquiries can be directed to the corresponding author.

## Author Contributions

MI conceived and designed the study. IP and MB performed study selection, data extraction, and interpreted the data. MI, IP, RP, and MB performed extensive search of relevant topics. IP and RP performed statistical analysis. MP, HG, MA, and AK performed review and extensive editing of the manuscript. All authors contributed significantly to the writing of the manuscript and approved the final manuscript.

## Conflict of Interest

The authors declare that the research was conducted in the absence of any commercial or financial relationships that could be construed as a potential conflict of interest.

## Publisher's Note

All claims expressed in this article are solely those of the authors and do not necessarily represent those of their affiliated organizations, or those of the publisher, the editors and the reviewers. Any product that may be evaluated in this article, or claim that may be made by its manufacturer, is not guaranteed or endorsed by the publisher.
