# Supplementary material for: Electrocardiographic Markers Indicating Right Ventricular Outflow Tract Conduction Delay as a Predictor of Major Arrhythmic Events in Patients With Brugada Syndrome: A Systematic Review and Meta-Analysis
Source: Front Cardiovasc Med. 2022 Jun 17;9:931622. doi: 10.3389/fcvm.2022.931622 (PMC9247269; doi:10.3389/fcvm.2022.931622)
Supplement: Supplementary file 1 [file Table_1.DOCX]

**Table S1.** Assessment of the risk of bias of the included studies using Newcastle Ottawa Scale (NOS).

| **No** | **Authors (Year)** | Exposed truly representative | Selection of non-exposed from the same community | Exposure ascertained by secure record or interview | Demonstration of outcome of interest not present at the start of the study | Study controls for other variables | Follow up long enough for outcome to occur | Complete follow up of all subjects accounted | Subject lost to follow up unlikely to introduce bias | Score | Risk of bias |
| --- | --- | --- | --- | --- | --- | --- | --- | --- | --- | --- | --- |
| 1 | Bigi (2007) | 1 | 1 | 1 | 1 | 0 | 1 | 1 | 1 | 7 | Low |
| 2 | Calo (2016) | 1 | 1 | 1 | 1 | 1 | 1 | 1 | 1 | 8 | Low |
| 3 | Ragab (1) (2017) | 1 | 1 | 1 | 0 | 2 | 1 | 1 | 1 | 8 | Low |
| 4 | Ragab (2) (2018) | 1 | 1 | 1 | 0 | 2 | 1 | 1 | 1 | 8 | Low |
| 5 | Morita (2018) | 1 | 1 | 1 | 1 | 0 | 1 | 1 | 1 | 7 | Low |
| 6 | Rizal (2019) | 1 | 1 | 1 | 1 | 0 | 1 | 1 | 1 | 7 | Low |
| 7 | Honarbakhsh (2021) | 1 | 1 | 1 | 0 | 2 | 1 | 1 | 1 | 8 | Low |
| 8 | Nagase (2018) | 1 | 1 | 1 | 0 | 2 | 1 | 1 | 1 | 8 | Low |
| 9 | Shinohara (2021) | 1 | 1 | 1 | 1 | 0 | 1 | 1 | 1 | 7 | Low |
| 10 | Michowitz (2019) | 1 | 1 | 1 | 1 | 0 | 1 | 1 | 1 | 7 | Low |
| 11 | Migliore (2018) | 1 | 1 | 1 | 0 | 2 | 1 | 1 | 1 | 8 | Low |
